# PTPN13 Participates in the Regulation of Epithelial–Mesenchymal Transition and Platinum Sensitivity in High-Grade Serous Ovarian Carcinoma Cells

**DOI:** 10.3390/ijms242015413

**Published:** 2023-10-21

**Authors:** Leticia Aptecar, Carole Puech, Evelyne Lopez-Crapez, Marion Peter, Peter Coopman, Véronique D’Hondt, Gilles Freiss

**Affiliations:** 1 IRCM (Institut de Recherche en Cancérologie de Montpellier), University of Montpellier, Inserm, ICM (Institut du Cancer de Montpellier), F-34000 Montpellier, Franceevelyne.crapez@icm.unicancer.fr (E.L.-C.); 2 CNRS—Centre National de la Recherche Scientifique, 1919 Route de Mende, F-34293 Montpellier, France; marion.peter@inserm.fr (M.P.); peter.coopman@inserm.fr (P.C.)

**Keywords:** HGSOC, PTPN13, EMT, platinum, drug sensitivity, cell aggressiveness

## Abstract

Epithelial ovarian cancer (EOC) is the leading cause of death from gynecological cancers in Western countries. High-Grade Serous Ovarian Carcinoma (HGSOC) accounts for 60–70% of EOC and is the most aggressive subtype. Reduced PTPN13 expression levels have been previously correlated with worse prognosis in HGSOC. However, PTPN13’s exact role and mechanism of action in these tumors remained to be investigated. To elucidate PTPN13’s role in HGSOC aggressiveness, we used isogenic PTPN13-overexpressing clones of the OVCAR-8 cell line, which poorly expresses PTPN13, and also PTPN13 CRISPR/Cas9-mediated knockout/knockdown clones of the KURAMOCHI cell line, which strongly expresses PTPN13. We investigated their migratory and invasive capacity using a wound healing assay, their mesenchymal-epithelial transition (EMT) status using microscopy and RT-qPCR, and their sensitivity to chemotherapeutic drugs used for HGSOC. We found that (i) PTPN13 knockout/knockdown increased migration and invasion in KURAMOCHI cells that also displayed a more mesenchymal phenotype and increased expression of the SLUG, SNAIL, ZEB-1, and ZEB-2 EMT master genes; and (ii) PTPN13 expression increased the platinum sensitivity of HGSOC cells. These results suggest that PTPN13 might be a predictive marker of response to platinum salts in HGSOC.

## 1. Introduction

Epithelial ovarian cancer is the leading cause of death in women with gynecological cancers in Western countries [1]. Most ovarian cancers are diagnosed at an advanced stage and, despite a standard treatment that includes cytoreductive surgery and systemic therapy, most patients will relapse and die from their disease. Recently, the addition of poly (ADP-ribose) polymerase (PARP) inhibitors as maintenance therapy after first-line treatment has significantly improved the patient prognosis [2,3,4,5]. The term ovarian carcinoma defines a heterogeneous group of epithelial tumors with different subtypes, pathophysiology, prognosis, and response to treatment. High-Grade Serous Ovarian Carcinoma (HGSOC) accounts for 60–70% of all ovarian carcinomas [6] and is the most aggressive subtype [7].

At relapse time, the disease is most often incurable. It is categorized as platinum-sensitive if the relapse occurs more than 6 months after the platinum-based treatment end, or platinum-resistant if the relapse occurs within 6 months of the treatment end [8]. The only identified biological predictor (biomarker) of HGSOC response to standard chemotherapy is the BRCA gene mutation status. BRCA, predisposition genes for ovarian and breast carcinoma, predict higher sensitivity to DNA-targeting chemotherapy drugs, including platinum agents [9]. BRCA mutations are found in 15% of patients with ovarian cancer and in ~25% of patients with HGSOC.

The PTPN13 gene is located at the chromosomal locus 4q21 [10] and encodes the type 13 non-receptor protein tyrosine phosphatase (PTPN13). We previously reported the first evidence that PTPN13 negatively regulates tumor growth in human breast cancer cell lines [11]. We showed that PTPN13 has a negative effect on cell survival via dephosphorylation of insulin receptor substrate 1 (IRS1), leading to PI3K inactivation [12]. We also demonstrated that PTPN13 mRNA expression is an independent prognostic marker of increased overall survival in breast cancer [13]. Other groups then confirmed and extended this result to hepatocellular carcinoma [14] and lung cancer [15]. Loss of heterozygosity (LOH) of chromosome 4q, where the PTPN13 locus is located, is observed in 67% of HGSOC [16]. Unlike the 17q LOH, the consequences of which have been largely attributed to the tumor suppressor TP53 located at 17p13, 4q LOH has not been clearly associated with the inactivation of potential tumor suppressor gene(s). In an initial pilot study, we found a correlation between reduced PTPN13 expression (measured by RT-PCR) and worse prognosis in a historical series of 28 HGSOC samples from patients treated with primary surgery and chemotherapy [17]. These results were subsequently confirmed by another group [18]. However, the exact role and mechanism of action of PTPN13 in these tumors remain to be investigated. Here, we used the OVCAR-8 (low PTPN13 expression) and KURAMOCHI (high PTPN13 expression) HGSOC cell lines to investigate PTPN13’s role in ovarian carcinogenesis, particularly in cancer cell mobility, invasiveness, and platinum sensitivity. We found that PTPN13 is involved in regulating cell mobility and invasiveness in HGSOC cells, and plays a role in their sensitivity to platinum salts.

## 2. Results

### 2.1. PTPN13 and HGSOC Cell Line Aggressivity

**Figure 1:**
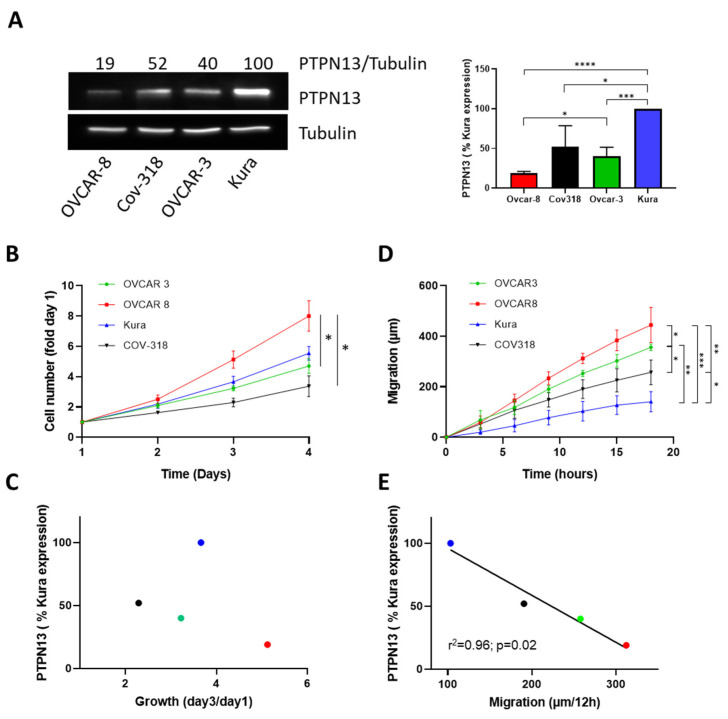
PTPN13 expression is correlated with cell migration. (**A**): PTPN13 expression in OVCAR-8, Cov-318, OVCAR-3, and KURAMOCHI (Kura) cells was monitored using western blotting using anti-PTPN13 antibodies. Equal loading was verified by re-probing with an anti-tubulin antibody. Left panel: representative western blot, the upper line shows the PTPN13/tubulin ratio; data are the mean of 4 independent experiments. Right panel: bar graph quantifying the western blot data; mean ± SD of 4 independent experiments. **** *p* < 0.0001, *** *p* < 0.001, * *p* < 0.05. Student’s *t*-test was used to compare means. (**B**): Cell growth of the indicated cell lines measured using the MTS assay. Results, expressed as fold of day 1, are the mean ± SD of three independent experiments. * *p* < 0.05. (**C**): PTPN13 expression level in OVCAR-8, COV-318, OVCAR-3, and KURAMOCHI cells in function of the cell line growth rate. (**D**): Directional migration of the indicated cell lines was assessed with the wound healing assay and monitored using video microscopy. Migration was expressed as the distance covered at each time point; mean ± SD of 5 independent experiments. *** *p* < 0.001, ** *p* < 0.01, * *p* < 0.05. (**E**): PTPN13 expression level in OVCAR-8, Cov-318, OVCAR-3, and KURAMOCHI cells as a function of the migration speed of that cell line. Two-way ANOVA performed with Prism (GraphPad software 8.0.1) was used for curve comparison. Correlations were evaluated with the Pearson test performed with Prism.

We carried out a preliminary analysis (PTPN13 expression, growth rate, and migratory capacity) in four representative HGSOC cell lines [19] available at the “SIRIC-Montpellier cancer” cell bank: COV-318, KURAMOCHI, OVCAR-3, and OVCAR-8 cells. All cell lines expressed PTPN13, but at very different levels. We observed the strongest expression in KURAMOCHI cells and the lowest expression in OVCAR-8 cells (19% of the KURAMOCHI cell levels). The other two cell lines showed intermediate expression levels (52% and 40% of the KURAMOCHI cell level for COV-318 and OVCAR-3 cells, respectively) (Figure 1A). OVCAR-8 cells (low PTPN13 level) displayed the fastest cell growth; however, cell growth was slower in COV-318 and OVCAR-3 cells (intermediate PTPN13 levels) than in KURAMOCHI cells (high PTPN13 level), thus failing to establish an obvious correlation between PTPN13 expression and cell proliferation (Figure 1B,C). The analysis of cell mobility using a wound healing assay showed that OVCAR-8 cells migrated rapidly (26 µm/h), followed by COV-318 and OVCAR-3 cells (15.9 and 21.4 µm/h, respectively) and then by KURAMOCHI cells (8.6 µm/h) (Figure 1D). This inverse correlation between PTPN13 expression and migratory capacity (r2 = 0.96) (Figure 1E) allows us to hypothesize a negative role for PTPN13 in the regulation of HGSOC cell migration. The use of siRNA directed against PTPN13 in the two cell lines most strongly expressing PTPN13, KURAMOCHI, and Cov-318 reinforces this hypothesis with a 50% increase in migration in these two cell lines when PTPN13 expression is inhibited (Supplementary Figure S1).

**Figure 2:**
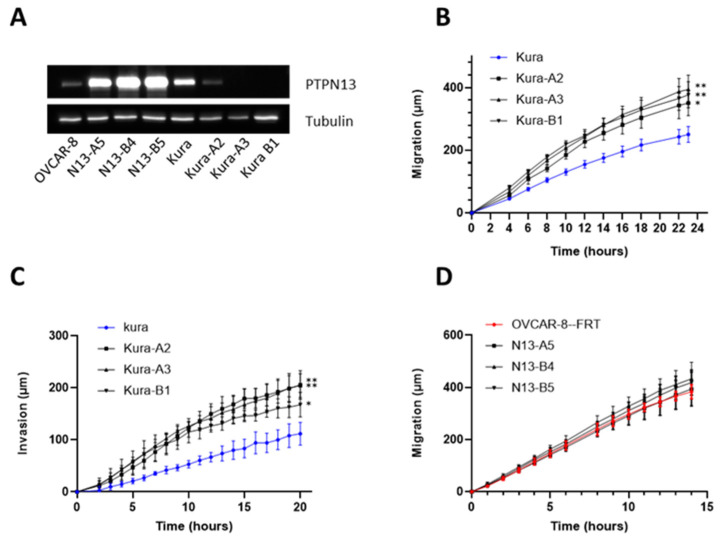
PTPN13 regulates KURAMOCHI cell motility and invasiveness. (**A**): PTPN13 expression in OVCAR8-FRT cells (OVCAR-8) and isogenic clones (N13-A5, N13-B4, and N13-B5) and in KURAMOCHI cells (Kura) and CRISPR/Cas9 clones (Kura-A2, Kura-A3 and Kura-B1) was monitored by western blotting using anti-PTPN13 antibodies. Equal loading was verified by re-probing with an anti-tubulin antibody. (**B**): Directional migration of KURAMOCHI cells (parental line and CRISPR/Cas9 clones) was assessed with the wound healing assay and monitored using video microscopy. Migration was expressed as the distance covered at each time point; mean ± SEM of 4 independent experiments. ** *p* < 0.01, * *p* < 0.05 versus parental KURAMOCHI cell line. (**C**): Invasiveness of KURAMOCHI cells (parental line and CRISPR/Cas9 clones) was evaluated with the wound healing assay and cells embedded in Matrigel and monitored using video microscopy. Invasiveness was expressed as the distance covered at each time point; mean ± SD of 4 independent experiments. ** *p* < 0.01, * *p* < 0.05 versus parental KURAMOCHI cells. (**D**): Directional migration of OVCAR-8 cells and the indicated clones was assessed with the wound healing assay monitored using video microscopy. Migration was expressed as the distance covered at each time point; mean ± SD of 5 independent experiments. Two-way ANOVA performed with Prism (GraphPad software 8.0.1) was used for comparing curves.

On the basis of these results, we chose the OVCAR-8 and KURAMOCHI cell lines to assess PTPN13’s role in HGSOC aggressiveness. Indeed, the OVCAR-8 cell line (low PTPN13 expression) had an aggressive profile (rapid proliferation and migration), but not the KURAMOCHI cell line that strongly expressed PTPN13. We developed isogenic clones of OVCAR-8 cells that overexpress PTPN13 using the Flp-In system, and KURAMOCHI cell clones that do not express PTPN13 using the CRISPR-Cas9 method (Figure 2A).

Knockout (KO)(Kura-A3 and Kura-B1) or knockdown (KD)(Kura-A2) of PTPN13 in KURAMOCHI cells did not modify cell growth (Supplementary Figure S2A), but significantly increased cell migration and invasiveness, two biological parameters associated with tumor cell aggressiveness (Figure 2B,C). Cell migration analysis using the wound healing assay showed that strong PTPN13 downregulation (Kura-A2 clone) and complete PTPN13 KO (Kura-A3 and Kura-B1 clones) significantly increased cell migration by 41%, 58%, and 68%, respectively, compared with parental KURAMOCHI cells (Figure 2B). Cell invasion analysis also revealed that partial (Kura-A2 clone) and complete PTPN13 silencing (Kura-A3 and Kura-B1 clones) significantly increased cell migration through Matrigel by 130%, 137%, and 116%, respectively, compared with parental KURAMOCHI cells (Figure 2C). This significant increase in cell invasion from KD or KO of PTPN13 clones was confirmed with the Boyden chamber assay (Supplementary Figure S3).

Overexpression of PTPN13 in OVCAR-8 cells did not modify cell growth (Supplementary Figure S2B) and mobility (Figure 2D) compared with OVCAR8-FRT control cells (transfected only with the pFRTLacZeo vector). 

This indicated that although PTPN13 contributes to KURAMOCHI cell motility inhibition, its overexpression is not sufficient to inhibit the motility of more aggressive HGSOC cell lines, such as OVCAR-8 cells. Indeed, OVCAR-8 cells, which are genetically less representative of HGSOCs than KURAMOCHI cells [19], are more tumorigenic [20], migrate faster [21] (and Figure 1D), are more proliferative [21] (and Figure 1B), and display more advanced epithelial–mesenchymal transition (EMT) characteristics [21].

**Figure 3:**
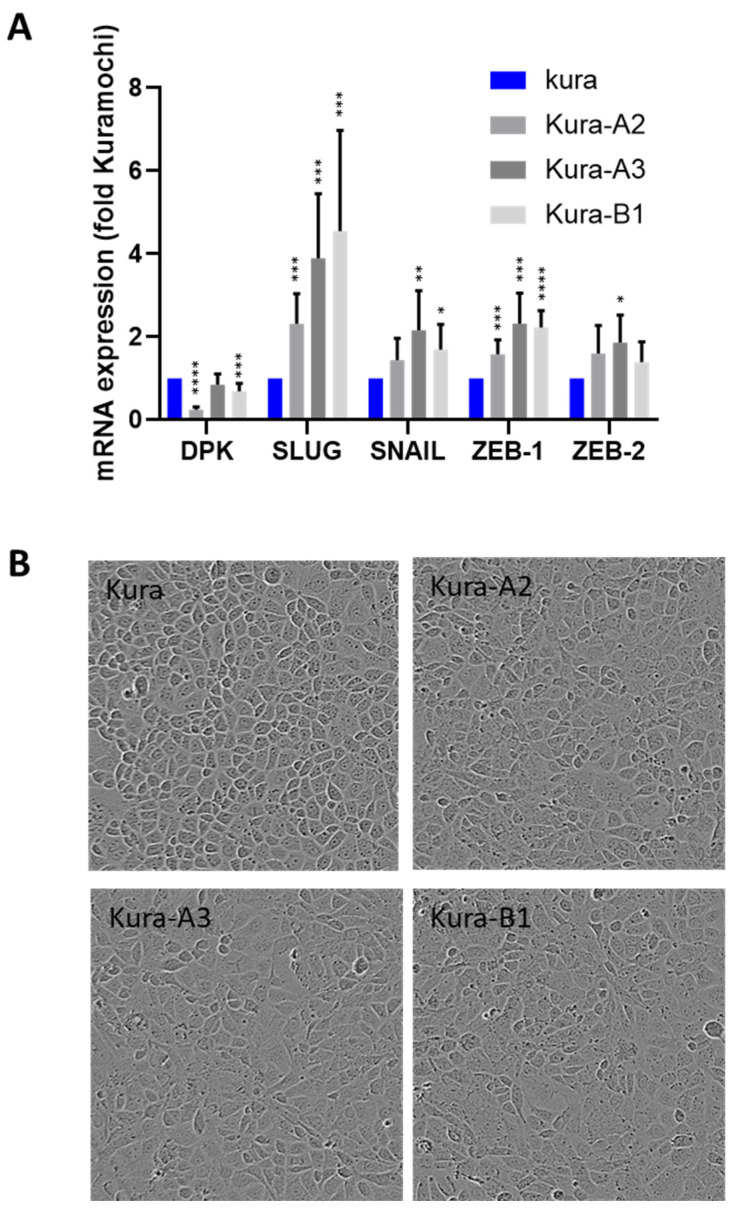
PTPN13 inhibits EMT in KURAMOCHI cells. (**A**): Expression of EMT master genes evaluated using RT-qPCR. Data were normalized to HPRT levels and are the mean ± SD relative to the mRNA levels in parental KURAMOCHI cells (Kura). N ≥ 5 experiments, * *p* < 0.05, ** *p* < 0.01, *** *p* < 0.001 and **** *p* < 0.0001 versus parental KURAMOCHI cells. The two-sided Student’s *t*-test was used to compare the two groups. (**B**): Phase-contrast microscopy images showing the morphology of parental KURAMOCHI cells (Kura) and clones in which PTPN13 was KD (Kura-A2) or KO (Kura-A3 and Kura-B1) (magnification: ×10).

As cell migration and invasiveness are associated with epithelial–mesenchymal transition (EMT), we used RT-qPCR to measure the expression of four transcription factors that drive EMT (SLUG, SNAIL, ZEB-1, and ZEB-2) and of desmoplakin (DPK), an epithelial cell–cell junction marker. PTPN13 KO and KD in KURAMOCHI cells, which enhanced migration and invasion, also moderately increased the expression of EMT driver genes and decreased DPK expression. Compared with parental KURAMOCHI cells, the expression levels of SLUG, ZEB-1, SNAIL, and ZEB-2 were increased by 2.3 to 4.5 times, by 1.6 to 2.3 times, by 1.4 to 2.2 times, and by 1.4 to 1.8 times, respectively, in the Kura-A2, Kura-A3, and Kura-B1 clones. Conversely, DPK expression was decreased by 1.2 to 4 times (Figure 3A).

**Figure 4:**
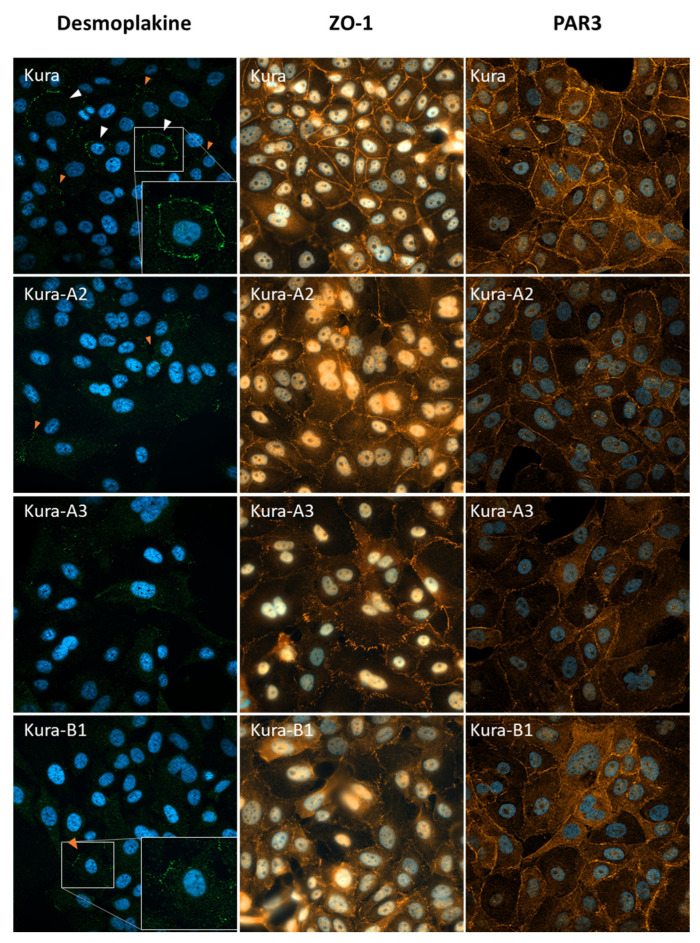
PTPN13 KO in KURAMOCHI cells modifies cell junction marker expression or localization: desmosomes were visualized with immunofluorescence analysis using anti-desmoplakin I + II antibodies (magnification: ×40), tight junctions were visualized with immunofluorescence analysis using anti -ZO-1, and the PAR3 polarity protein was visualized with immunofluorescence analysis (magnification: ×63); nuclei were counterstained with Hoechst. Arrows show cells with total (white) or partial (orange) cell junction localization of desmoplakin.

Correspondingly, phase contrast microscopy showed that parental KURAMOCHI cells displayed an epidermoid phenotype that progressively disappeared in the clones with PTPN13 KO/KD (Figure 3B). We then studied desmoplakin expression and localization using immunofluorescence (Figure 4). Conversely, in some parental KURAMOCHI cells, desmoplakin was localized (Figure 4, white arrows) or partially localized (Figure 4, orange arrows) at intercellular junctions. This intercellular localization decreased in the KURAMOCHI clones in which PTPN13 was KD or KO. Furthermore, the localization of the tight junction marker, ZO-1 [22], showed a clear localization along cell junctions in parental KURAMOCHI cells and a more diffuse and punctate localization in KO or KD clones for PTPN13 (Figure 4). Finally, in line with a loss of cell polarity, the PAR3 polarity protein ([23]) expression at cell junctions is strongly inhibited by the loss of PTPN13 expression (Figure 4). Altogether, these results demonstrated that OVCAR-8 cells have already undergone EMT, whereas KURAMOCHI cells exhibit a partial epidermoid phenotype that is lost upon PTPN13 expression silencing.

### 2.2. PTPN13 and HGSOC Cell Line Sensitivity to Chemotherapy Treatments

Then, to validate our initial results on PTPN13 prognostic and predictive value [17], we assessed PTPN13 expression with digital droplet PCR (ddPCR) using RNA extracted from tumor sections of a new series of 60 HGSOC samples for which follow-up data (recurrence) were available. After dividing the tumor samples into two groups based on PTPN13 expression (cut-off lower tercile), we observed a non-significant trend towards longer recurrence-free survival in patients with high PTPN13 expression (HR = 1.27; *p* = 0.33) (Supplementary Figure S4). As this new analysis did not confirm PTPN13’s prognostic value, we compared the characteristics of patients/tumors included in this new series and in the previous series. The patients in the previous study, analyzed with RT/PCR, all treated before the year 2000, had predominantly received first-line treatment with platinum salts alone or combined with cyclophosphamide (only four patients were treated with paclitaxel combined with platinum salts). Conversely, in the new series, all patients received the carboplatin/paclitaxel combination. Therefore, we examined PTPN13’s effect on the OVCAR-8 and KURAMOCHI cell sensitivity to platinum agents and paclitaxel.

**Figure 5:**
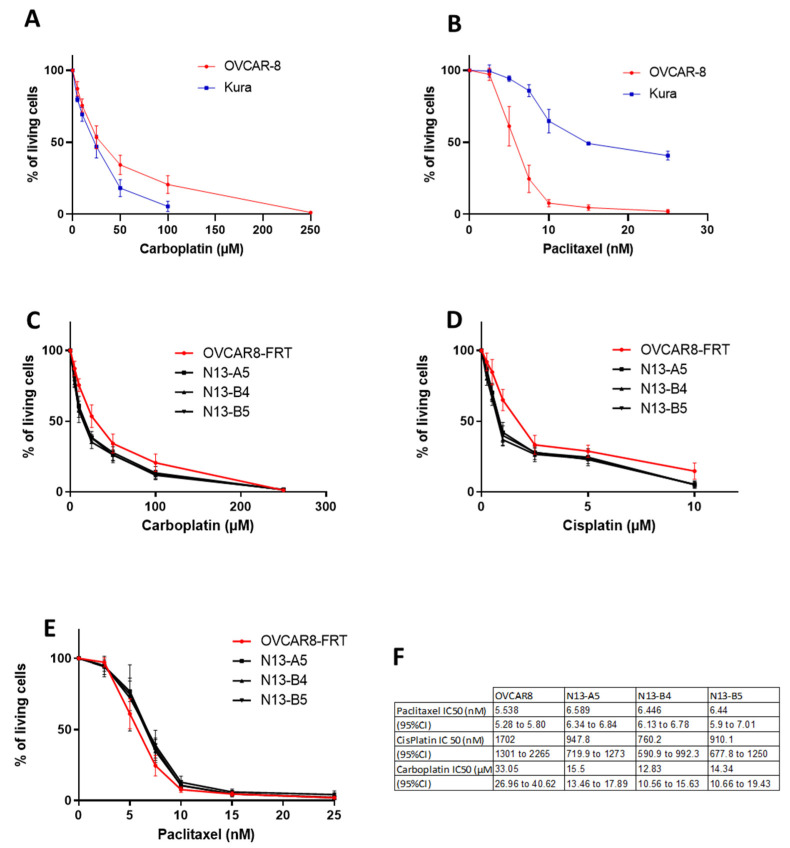
PTPN13 overexpression increases OVCAR-8 cell sensitivity to platinum agents. (**A**): Comparison of carboplatin sensitivity (percentage of living cells at the indicated concentration) in OVCAR-8-FRT and KURAMOCHI (Kura) cells. Mean ± SD of 5 (Kura) and 7 (OVCAR-8) independent experiments. *p* < 0.0001. (**B**): Comparison of paclitaxel sensitivity in OVCAR-8-FRT and KURAMOCHI (Kura) cells. Mean ± SD of 5 (Kura) and 9 (OVCAR-8) independent experiments. *p* < 0.0001. (**C**): Comparison of carboplatin sensitivity in OVCAR-8-FRT cells and clones that overexpress PTPN13. Mean ± SD of 7 (OVCAR-8) and 5 (N13 clones) independent experiments. *p* < 0.0001 for each clone compared to OVCAR-8-FRT. (**D**): Comparison of cisplatin sensitivity in OVCAR-8-FRT cells and clones that overexpress PTPN13. Mean ± SD of 7 (OVCAR-8) and 5 (N13 clones) independent experiments. *p* < 0.0001 for each clone compared to OVCAR-8-FRT. (**E**): Comparison of paclitaxel sensitivity in OVCAR-8-FRT cells and clones that overexpress PTPN13. Mean ± SD of 9 (OVCAR-8) and 6 (N13 clones) independent experiments. *p* < 0.0001 for N13-A5 versus OVCAR-8-FRT, *p* < 0.01 for N13-B4 and N13-B5 versus OVCAR-8-FRT cells. (**F**): Table showing the IC50 for the indicated chemotherapeutic agents in OVCAR-8-FRT cells and clones that overexpress PTPN13. Curves were compared with a two-way ANOVA performed with Prism (GraphPad software 8.0.1).

We first compared the OVCAR-8 and KURAMOCHI cell sensitivity to carboplatin and paclitaxel. OVCAR-8 cells (low PTPN13 expression) were less sensitive to carboplatin (IC50 = 33.1 µM; 95% CI: 27–40.6) than KURAMOCHI cells (high PTPN13 expression) (IC50 = 16.05 µM; 95% CI: 12.4–20.7) (Figure 5A). Conversely, KURAMOCHI cells were less sensitive to paclitaxel (IC50 = 9.5 nM; 95% CI: 8.86–10.13) than OVCAR-8 cells (IC50 = 5.5 nM; 95% CI: 5.3–5.8) (Figure 5B). To determine whether these differences in sensitivity were due to differences in PTPN13 expression, we studied the effect of PTPN13 overexpression or silencing on chemotherapy sensitivity. In the isogenic clones of OVCAR-8 cells, PTPN13 overexpression increased sensitivity to carboplatin and cisplatin, while it weakly, but significantly, decreased sensitivity to paclitaxel (Figure 5C–F). For carboplatin, the IC50 decreased from 33.05 µM (95% CI: 26.7–40.6) in OVCAR-8-FRT cells to 15.5 µM (95% CI: 13.5–17.9), 12.83 µM (95% CI: 10.6–15.6), and 14.34 µM (95% CI: 10.7–19.4) in the N13-A5, N13-B4, and N13-B5 clones, respectively. The cisplatin IC50 decreased from 1.7 µM (95% CI: 1301–2265) in OVCAR-8-FRT cells to 947.8 nM (95% CI: 720–1273), 760.2 nM (95% CI: 591–992), and 910.1 nM (95% CI:678–1250) in the N13-A5, N13-B4, and N13-B5 clones, respectively. Conversely, the IC50 for paclitaxel increased from 5.538 nM (95% CI: 5.28–5.8) in OVCAR-8-FRT cells to 6.59 nM (95% CI: 6.34–6.84), 6.45 nM (95% CI: 6.13–6.78), and 6.44 nM (95% CI: 5.9–7.1) in the N13-A5, N13-B4, and N13-B5 clones, respectively. 

**Figure 6:**
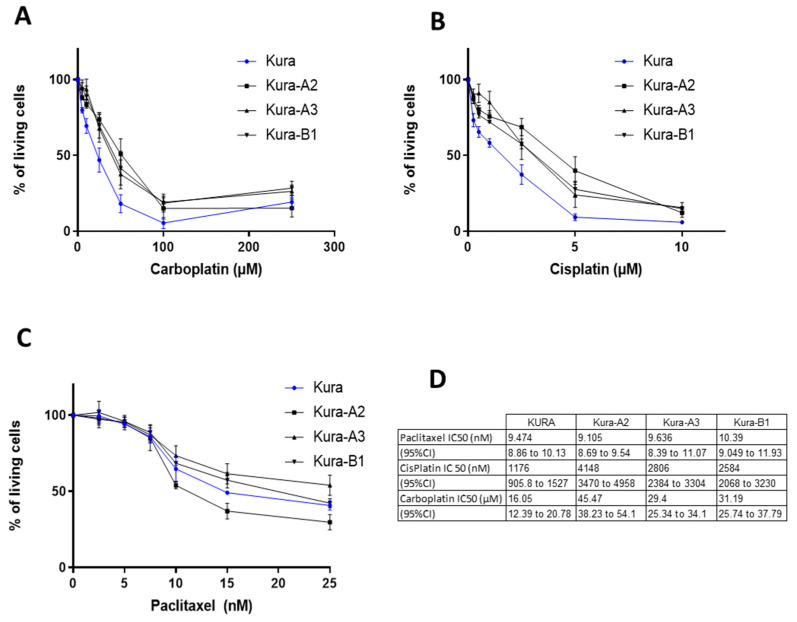
PTPN13 silencing decreases KURAMOCHI cell sensitivity to platinum agents. (**A**): Comparison of carboplatin sensitivity (percentage of living cells at the indicated concentration) in parental KURAMOCHI (Kura) cells and PTPN13 KO or KD clones. Mean ± SD of 5 (Kura and Kura-A2) and 4 (Kura-A3 and Kura-B1) independent experiments. *p* < 0.0001 for the Kura A2 clone versus Kura cells, *p* < 0.001 for the Kura-A3 and Kura-B1 clones vs. Kura cells. (**B**): Comparison of cisplatin sensitivity in parental KURAMOCHI cells and PTPN13 KO or KD clones. Mean ± SD of 5 (Kura and Kura-A2) and 4 (Kura-A3 and Kura-B1) independent experiments. *p* <0.0001 for the Kura A2 clone versus Kura cells, *p* < 0.005 for the Kura-A3 et Kura-B1 clones vs. Kura cells. (**C**): Comparison of paclitaxel sensitivity in parental KURAMOCHI cells and PTPN13 KO or KD clones. Mean ± SD of 5 (Kura and Kura-A2) or 4 (Kura-A3 and Kura-B1) independent experiments. (**D**): Table showing the IC50 for the indicated chemotherapeutic agents in parental KURAMOCHI cells and PTPN13 KO or KD clones. Curves were compared with a two-way ANOVA performed with Prism (GraphPad software 8.0.1).

On the other hand, PTPN13 KO/KD reduced the sensitivity of KURAMOCHI cells to platinum salts. For carboplatin, the IC50 increased from 16.05 µM (95% CI: 12.4–20.8) in parental cells to 45.5 µM (95% CI: 38.2–54.1), 29.4 µM (95% CI: 25.3–34.1), and 31.2 µM (95% CI: 25.7–37.79) in the Kura-A2, Kura-A3, and Kura-B1 clones, respectively (Figure 6A–D). The cisplatin IC50 increased from 1.18 µM (95% CI: 0.9–1.5) in parental cells to 4.15 µM (95% CI: 3.47–4.96), 2.8 µM (95% CI: 2.38–3.3), and 2.58 µM (95% CI: 2.07–3.23) in the Kura-A2, Kura-A3, and Kura-B1 clones, respectively. Consistent with this sensitizing effect of PTPN13 to platinum salt-induced apoptosis, we observed higher PARP expression in wild-type OVCAR-8 cells than in N13-B4 cells overexpressing PTPN13. Moreover, this low expression was strongly and rapidly inhibited by carboplatin in cells overexpressing PTPN13. On the other hand, wild-type KURAMOCHI cells expressed less PARP than Kura-B1 (KO for PTPN13) cells, and we also observed a rapid PARP degradation in wild-type cells (supplementary Figure S5).

We did not observe any significant difference in paclitaxel responsiveness between parental KURAMOCHI cells and the KO/KD clones. 

Altogether, our results, obtained in two HGSOC cell lines, demonstrated that PTPN13 positively regulates the sensitivity of HGSOC cells to platinum salts, whereas it has little or no effect on paclitaxel sensitivity. 

## 3. Discussion

PTPN13 expression is a prognostic marker in many tumor types [13,14,15], including HGSOC [17,18], but its mechanism of action in HGSOC had not been studied. Therefore, here, we silenced PTPN13 in the KURAMOCHI cell line (high PTPN13 expression and low aggressivity) and overexpressed PTPN13 in the OVCAR-8 cell line (weak PTPN13 expression and higher aggressivity). In KURAMOCHI cells, PTPN13 KO/KD increased cell migration and invasiveness, indicating a role in negatively regulating the aggressiveness of these cells. PTPN13 KO/KD also led to increased expression of EMT master genes (SNAIL, SLUG, ZEB1, and 2), decreased expression and intercellular junction localization of cell junction markers such as desmoplakin, ZO-1, and PAR3, and a morphological shift towards a mesenchymal phenotype. PTPN13 overexpression in OVCAR-8 cells did not modify migration or EMT driver gene expression. This suggests that although PTPN13 participates in the inhibition of KURAMOCHI cell motility, its overexpression is not sufficient to inhibit the migration of more aggressive cell lines, such as OVCAR-8 cells that already have an established mesenchymal phenotype. This inhibitory effect of PTPN13 on cell migration and invasion is consistent with the observations in other tumor types (for review, see [24]). PTPN13 overexpression in a hepatocellular carcinoma cell line [14] and PTPN13 inhibition in lung cancer cells [25] and human umbilical endothelial cells [26] may regulate EMT driver gene expression. In addition, PTPN13 might also inhibit EMT through the stabilization of intercellular junctions via its positive role in desmosome formation [27]. High expression of PTPN13 in induced retinal pigment epithelial cells endows them with an epithelial-to-mesenchymal transition-resistant capacity through dephosphorylating syntenin1, and subsequently promotes the internalization and degradation of transforming growth factor-β receptors [28]. Inhibition of miRNA-200b, which targets PTPN13, is associated with EMT [29]. PTPN13 downregulation also increases PC3 cell (prostate cancer) invasion through the Matrigel-coated membrane and upregulates invasion-related genes [30].

Here, we demonstrated that PTPN13 overexpression, in isogenic clones of OVCAR-8 cells, increases their sensitivity to carboplatin and cisplatin, as indicated by the 2-fold decrease in the IC50 value. We also observed this PTPN13 sensitizing effect in KURAMOCHI cells in which PTPN13 silencing decreased their sensitivity to platinum salts (2–3-fold increase in IC50, depending on the clone). PTPN13’s role in the responsiveness to cancer therapies appears to be highly dependent on the tumor type. As PTPN13 induces resistance to FAS-mediated apoptosis [31], several studies evaluated whether resistance to cancer treatment is associated with PTPN13 expression (for review, [24]). However, few studies investigated the relationship between PTPN13 expression and sensitivity to platinum salts. In colon cancer cells, where the FAS receptor is strongly expressed [32], a study showed that PTPN13 silencing with siRNA improves their sensitivity to oxaliplatin by increasing FAS-induced apoptosis [33]. In line with these observations, inhibition of the PTPN13FAS interaction with the SLV peptide in PTPN13-overexpressing CD133-positive colon cancer stem cells increases their responsiveness to oxaliplatin, restoring FAS-induced apoptosis [34]. Conversely, another study demonstrated PTPN13’s positive involvement in cisplatin sensitivity of head and neck squamous cell carcinoma (WSU-HN6 and CAL-27) cell lines [35]. 

Several mechanisms may explain PTPN13’s effect on platinum salt sensitivity. EMT, which is induced by PTPN13 KO/KD in our model, plays an important role in platinum salt resistance in various tumor types (for review, [36,37]), including ovarian cancer (for review, [38]). Inhibition of anti-apoptotic transduction pathways by PTPN13 [12,35] also could explain this sensitizing effect. VCP, a PTPN13 substrate [39], plays an important role in the regulation of DNA damage repair [40,41], although the effect of its dephosphorylation by PTPN13 remains unknown (for review, [42]). More generally, the importance of tyrosine phosphatases in regulating DNA damage repair received little attention, whereas the importance of tyrosine phosphorylation appears to be increasing [43,44,45,46]. 

In summary, using loss- and gain-of-expression approaches, we demonstrated that PTPN13 acts as a tumor suppressor in HGSOC cell lines; it inhibits tumor aggressiveness by regulating SNAIL, SLUG, and ZEB-dependent EMT, and also sensitizes these cell lines to platinum salts. Additional studies are now required to determine the fine mechanism of this platinum sensitization and to define the value of PTPN13 as a predictive marker of response to platinum salts and to therapies targeting DNA damage repair, such as PARP inhibitors.

## 4. Materials and Methods

### 4.1. Cell Lines and Antibodies

The NIH-OVCAR-3 and -8, KURAMOCHI, and COV-318 cell lines were obtained from the “SIRIC-Montpellier cancer” cell bank. NIH-OVCAR-3 and -8 and KURAMOCHI cells were cultured in RPMI and COV-318 cells in DMEM, all supplemented with 10% fetal bovine serum (FBS).

The Flp-In OVCAR-8 clones that contain a unique Flp recombination target (FRT) site were obtained using stable transfection of the pFRTLacZeo vector (Invitrogen) and selection with 100 µg/mL zeocin. One clone with a unique FRT site insertion was selected as OVCAR-8-FRT. Flp-In OVCAR-8 clones that express PTPN13 were generated following the manufacturer’s instructions. Briefly, HA-tagged PTPN13 [12] was cloned in the pcDNA5/FRT vector (Invitrogen) to generate the pcDNA5/FRT/PTPN13 plasmid. pcDNA5/FRT/PTPN13 and pOG44 (Invitrogen) were co-transfected, at a 1:9 (*w*/*w*) ratio, in Flp-In OVCAR-8 cells (OVCAR-8-FRT), and clones resistant to hygromycin B (500 µg/mL) were selected. PTPN13 expression was confirmed in three selected clones (N13-A5, N13-B4, and N13-B5).

PTPN13 KD or KO KURAMOCHI clones using CRISPR-Cas9 were obtained by co-transfection of KURAMOCHI cells with the pSpCAS9wt-t2a-GFP (encoding the CRISPR-associated endonuclease Cas9) and U6-gRNA:PGK-puro-2A-tagGFP (encoding a guide RNA targeting PTPN13: GAAGAATGAGGATAACCGAAGG, gRNA1, or AAGGGCAGCAGGATCAGCTAGG, gRNA2) plasmids and the puromycin resistance gene (Montpellier Genomic Collection). Clones resistant to puromycin (2 µg/mL) were selected. Loss of PTPN13 expression was confirmed in three selected clones (Kura-A2, Kura-A3 with gRNA2, and Kura-B1 with gRNA1). 

The following monoclonal and polyclonal primary antibodies were used: anti-tubulin (clone B-5-1-2, Sigma), anti-PTPN13 (25944-1 AP, Proteintech), anti-PAR3 (07-330, Millipore), anti-ZO-1 (61-7300, Invitrogen), and anti-desmoplakin I + II (Ab16434, Abcam). HRP-labeled horse anti-mouse IgG (7076, Cell signaling technology) and HRP-labeled goat anti-rabbit IgG (7074, Cell signaling technology) were used as secondary antibodies for western blotting. Alexa Fluor™ Plus 488-labeled donkey anti-mouse IgG (H + L) (Invitrogen) was used as a secondary antibody for immunofluorescence analyses.

### 4.2. Western Blot Analysis

Cells were washed twice in ice-cold PBS and lysed in lysis buffer (40 mM Tris-HCl, pH8, 5 mM MgCl_2_, 40 mM Na4P207, 1% Triton X-100, 10 mM EDTA, 50 mM NaF, 100 µM Na3VO4, 1/25 protease inhibitor cocktail (ChemCruz)). Equal protein amounts of each lysate were separated on 7.5% SDS/PAGE gels before immunoblotting, as previously described [12], with the indicated antibodies.

### 4.3. Wound Healing Assay and Invasion Assay

For the wound healing assay, 60,000 cells (OVCAR-8, OVCAR-3, and COV-318) or 80,000 cells (KURAMOCHI) were seeded in 96-well plates. After 24 h, cell layers (at 90–100% of confluence) were wounded by scraping with the Incucyte^®^ Wound Maker 96-Tool (Sartorius). Cells were washed with a culture medium to remove floating cells and a fresh medium supplemented with 10% FBS was added. Wound healing was imaged with an Incucyte^®^ Live-Cell imager (Sartorius) every 2 h post-wounding. The wound width was evaluated in at least three wells using the Incucyte^®^ Cell Migration Scratch Wound Analysis software. For the invasion assay, Matrigel (300 µg/mL) (Becton Dickinson) was added after wounding and washing.

### 4.4. Cell Growth Assay

For the cell growth assay, 6000 cells/well were seeded in four 96-well plates. Living cells were quantified at 24 h post-seeding (day 1) and at days 2, 3, and 4 using the CellTiter 96^®^ Aqueous One Solution Cell Proliferation Assay (Promega). Cell growth was determined as the signal intensity at day 4, 3, or 2 divided by the signal at day 1.

### 4.5. Cell Survival Assay

For this assay, 2000 cells/well (OVCAR-8) or 4000 cells/well (KURAMOCHI) were seeded in 96-well microplates (Falcon Multiwell, Dutscher, 67170 Brumath, France), 24 h before any treatment. The indicated chemotherapeutic drugs in the fresh medium were then added for 72 h. Living cells were quantified using the CellTiter 96^®^ Aqueous One Solution Cell Proliferation Assay (Promega). Cell survival was expressed as the percentage relative to untreated cells.

### 4.6. Droplet-Based Digital PCR (ddPCR)

ddPCR was performed as described [47] using micro-dissected paraffin-embedded tumor sections [48] and the PTPN13 gene primers TGGTAAAACTGGTTCTGGGAAAT (forward) and TCAGGCTTTTGTTCACTGTTACA (reverse), and the probe TGGGACAGATCAGCTTTCC. RPP30 was used as a housekeeping gene.

### 4.7. Patients and Tumor Samples

HGSOC samples were selected among the primary cytoreductive surgery specimens stored at the Institut Régional du Cancer de Montpellier (ICM). At sampling, all patients were chemotherapy-naive, and were then treated with the carboplatin/paclitaxel combination as first-line systemic treatment. All samples were anonymized, and expression analyses were performed blinded to the clinical and pathological data. HGSOC and the tumor grade were defined according to the World Health Organization criteria. All samples were from archival blocks of paraffin-embedded tissue specimens. Informed consent was obtained from all subjects involved in the study.

### 4.8. Immunofluorescence Analysis

Cells plated on coverslips were fixed in methanol at −20 °C for 15 min, and permeabilized with 0.5% Triton X-100 in PBS for 10 min. Immunolabeling was performed as described [27]. Coverslips were mounted and images were taken with a Zeiss Imager M2 and a PlanApochromat 40x/1.3 DIC (oil) and 63x/1.4 (oil) objective (Zeiss) with apotome.

### 4.9. Quantitative RT-PCR

RNA was isolated using the Tri Reagent (Zymo Research) according to the manufacturer’s instructions, and quantified by measuring the absorbance at 260 nm. RNA quality was checked by assessing the ratio between the absorbance values at 260 and 280 nm. Total RNA (1 μg) was reverse transcribed using 5 μM of random hexamers (Roche) and Superscript II, according to the manufacturer’s instructions (Invitrogen). Quantitative real-time polymerase chain reaction (qPCR) analysis was performed using the amount of cDNA corresponding to 12.5 ng of total RNA on a Light Cycler 480 apparatus (Roche) with the Light Cycler FastStart DNA Master PLUS SYBR Green I Kit (Roche) according to the manufacturer’s instructions. The primers used for PCR amplification were ZEB-1 forward 5′ GTT CTG CCA ACA GTT GGT TT 3′, reverse 5′ GCT CAA GAC TGT AGT TGA TG 3′; ZEB-2 forward 5′ TCT GAA GAT GAA GAA GGC TG 3′, reverse 5′ AGT GAA TGA GCC TCA GGT AA 3′; SLUG forward 5′ CAG TGG CTC AGA AAG CCC C 3′, reverse 5′ TCA GCT TCA ATG GCA TGG G 3′; SNAIL forward 5′ GCT GCA GGA CTC TAA TCC AGA 3′, reverse 5′ GAC AGA GTC CCA GAT GAG CAT 3′; HPRT forward 5′ CTG ACC TGC TGG ATT ACA 3′, reverse 5′ GCG ACC TTG ACC ATC TTT 3′. The relative quantification of each gene expression was performed using the comparative cycle threshold (CT) method, with control cells as the calibrator sample (expression set at 1), and HPRT values as endogenous RNA normalization control [13].

## Data Availability

The data generated during and/or analyzed during the current study are available from the corresponding author upon reasonable request.

## Supplementary Materials

The supporting information can be downloaded at https://www.mdpi.com/article/10.3390/ijms242015413/s1
