# Supplementary material for: PTPN13 Participates in the Regulation of Epithelial–Mesenchymal Transition and Platinum Sensitivity in High-Grade Serous Ovarian Carcinoma Cells
Source: Int J Mol Sci. 2023 Oct 21;24(20):15413. doi: 10.3390/ijms242015413 (PMC10607604; doi:10.3390/ijms242015413)
Supplement: Supplementary file 1 [file ijms-24-15413-s001.zip › Supplementary Figure S1.pdf]

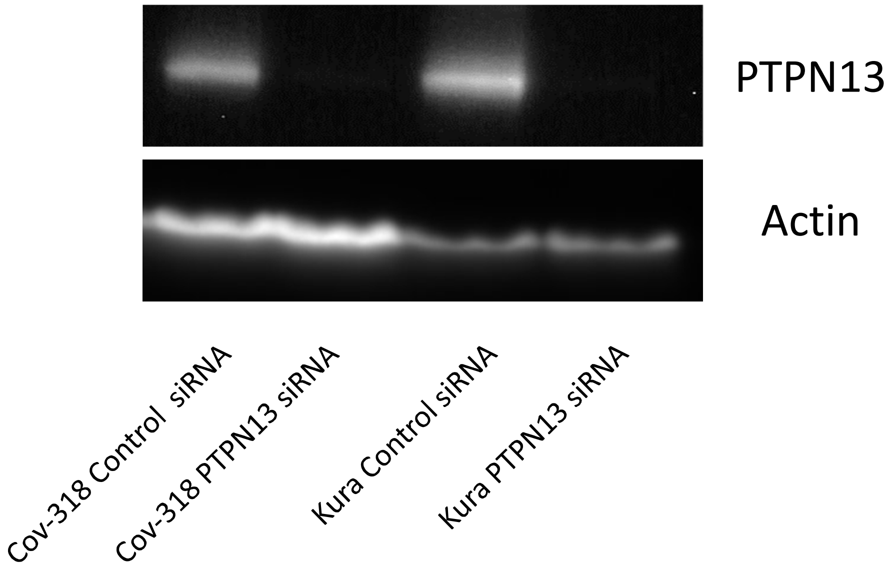

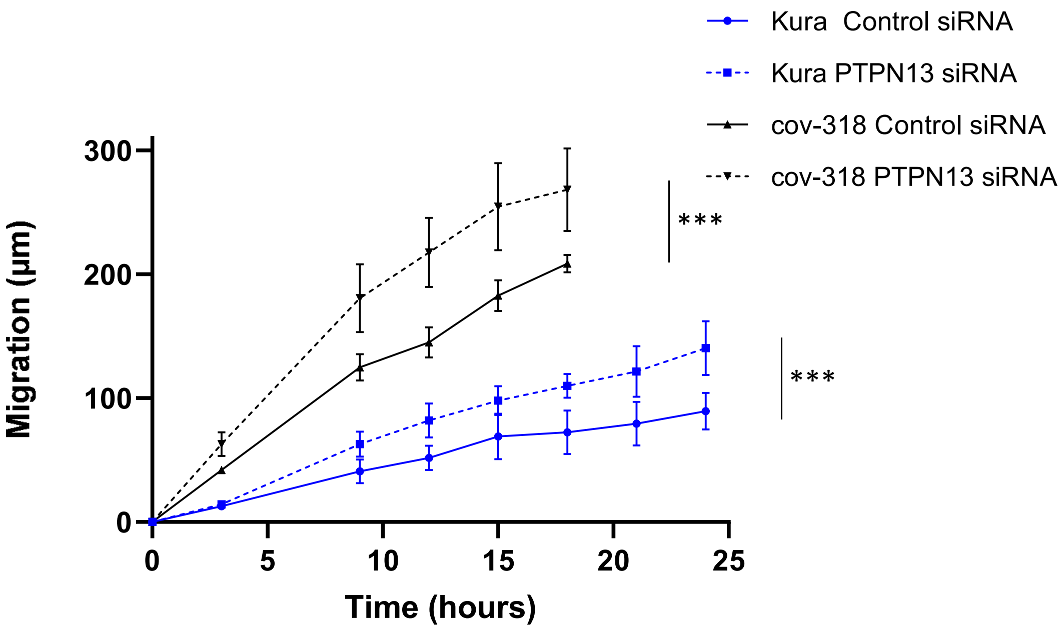

Supplementary Figure S1. Inhibition of PTPN13 expression by siRNA increases migration of HGSOC cell lines expressing high PTPN13 levels: Cov-318 and KURAMOCHI (Kura) cells were transfected with control siRNA (MWG) or siRNA against PTPN13 (CAGAUCAGCUUUCCUGUAA) using Interferin (Polyplus). A: PTPN13 expression was monitored by western blotting using anti-PTPN13 antibodies. Equal loading was verified by re-probing with an anti-actin antibody. B: Directional migration of the indicated cell lines was assessed with the wound healing assay and monitored by video microscopy. Migration was expressed as the distance covered at each time point; mean ± SD of 3 wells. ***P <0.001. Two-way ANOVA performed with Prism (GraphPad software) was used for curve comparison.
