# Supplementary material for: PTPN13 Participates in the Regulation of Epithelial–Mesenchymal Transition and Platinum Sensitivity in High-Grade Serous Ovarian Carcinoma Cells
Source: Int J Mol Sci. 2023 Oct 21;24(20):15413. doi: 10.3390/ijms242015413 (PMC10607604; doi:10.3390/ijms242015413)
Supplement: Supplementary file 1 [file ijms-24-15413-s001.zip › Supplementary Figure S2.pdf]

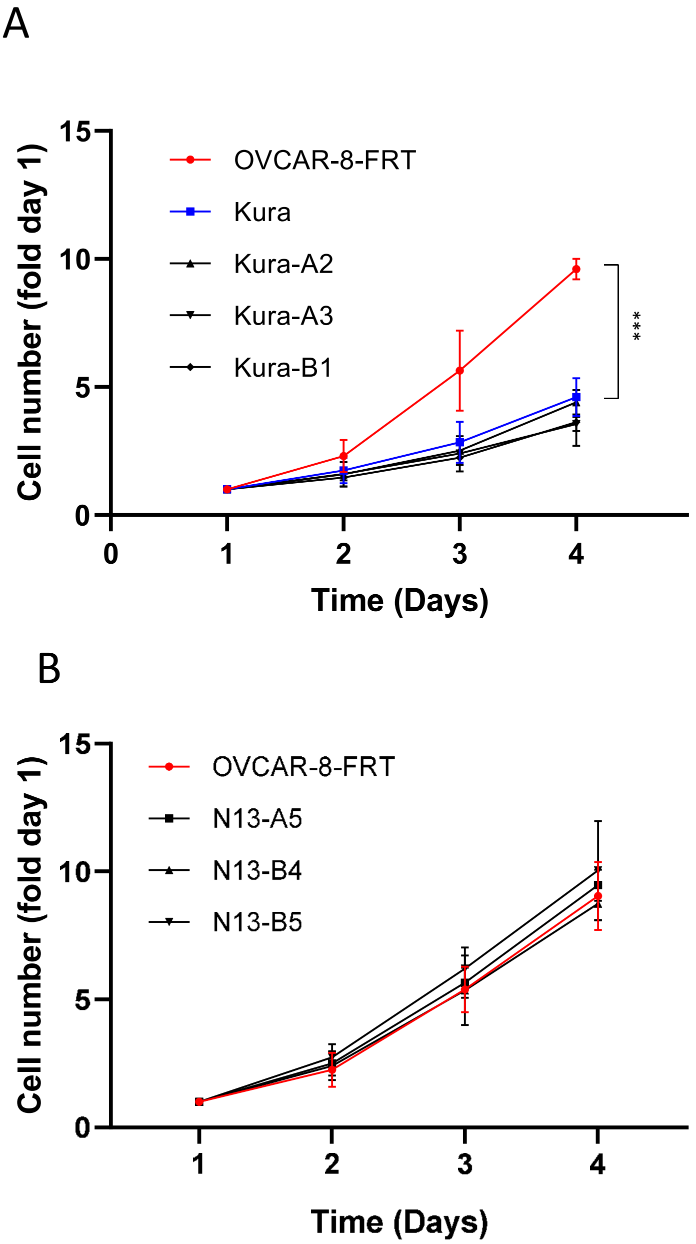

Supplementary Figure S2: PTPN13 is not involved in HGSOC cell growth regulation. Cell growth was measured using the MTS assay. Results are expressed as fold of Day 1 and are the mean ± SD of three independent experiments. **A**: Comparison of cell growth in the parental KURAMOCHI cell line (Kura), in three clones were PTPN13 was knocked down/out with the CRISPR/Cas9 method (Kura-A2, Kura-A3 and Kura-B1), and in OVCAR-8-FRT cells. ***P <0.001, **B**: Comparison of cell growth in OVCAR-8-FRT cells and three isogenic clones (N13-A5, N13-B4 and N13-B5) that overexpress PTPN13.
