# Supplementary material for: PTPN13 Participates in the Regulation of Epithelial–Mesenchymal Transition and Platinum Sensitivity in High-Grade Serous Ovarian Carcinoma Cells
Source: Int J Mol Sci. 2023 Oct 21;24(20):15413. doi: 10.3390/ijms242015413 (PMC10607604; doi:10.3390/ijms242015413)
Supplement: Supplementary file 1 [file ijms-24-15413-s001.zip › Supplementary Figure S3.pdf]

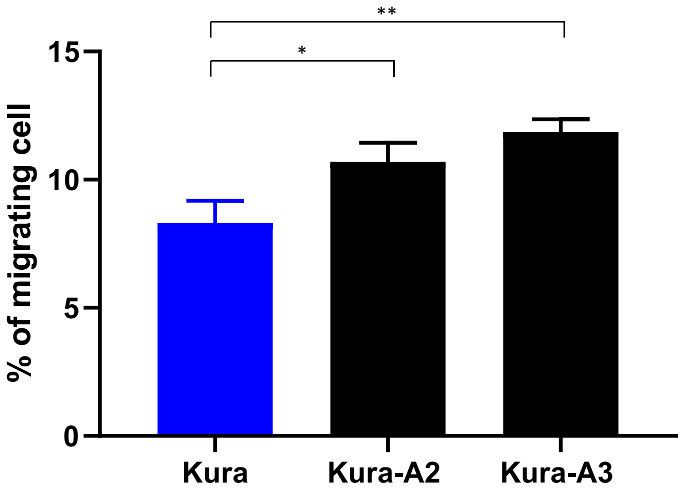

Supplementary Figure S3. PTPN13 regulates KURAMOCHI cell invasiveness: Invasiveness of KURAMOCHI cells (parental line and CRISPR/Cas9 clones; Kura-A2 and Kura-A3) was evaluated with Boyden chambers coated with 30µg Matrigel and quantified by the MTT assay [12]. Invasiveness was expressed as the percentage of cells migrating to the bottom chamber after 24h; mean ± SD of 3 Boyden chambers. **P <0.01, *P <0.05 versus parental KURAMOCHI cells. t-test performed with Prism (GraphPad software) was used for comparing the means.
