# Supplementary material for: PTPN13 Participates in the Regulation of Epithelial–Mesenchymal Transition and Platinum Sensitivity in High-Grade Serous Ovarian Carcinoma Cells
Source: Int J Mol Sci. 2023 Oct 21;24(20):15413. doi: 10.3390/ijms242015413 (PMC10607604; doi:10.3390/ijms242015413)
Supplement: Supplementary file 1 [file ijms-24-15413-s001.zip › Supplementary Figure S4.pdf]

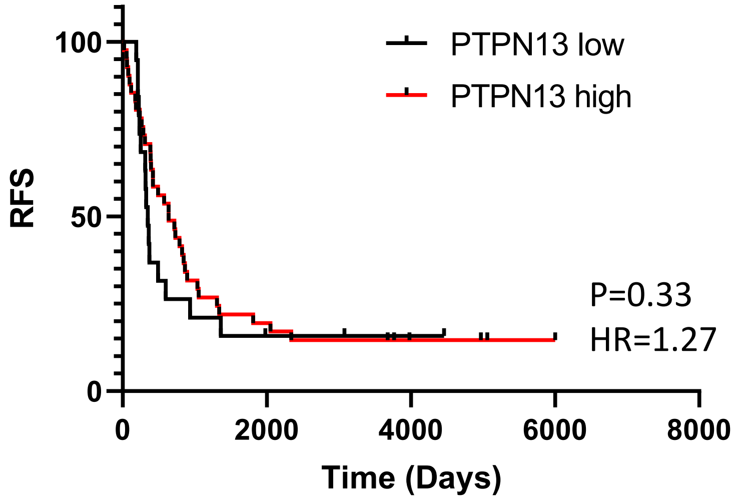

Supplementary Figure S4: Relapse-Free Survival in a cohort of patients (n=60) with HGSOC grouped according to *PTPN13* mRNA expression level assessed by RT-ddPCR.
