# Supplementary material for: PTPN13 Participates in the Regulation of Epithelial–Mesenchymal Transition and Platinum Sensitivity in High-Grade Serous Ovarian Carcinoma Cells
Source: Int J Mol Sci. 2023 Oct 21;24(20):15413. doi: 10.3390/ijms242015413 (PMC10607604; doi:10.3390/ijms242015413)
Supplement: Supplementary file 1 [file ijms-24-15413-s001.zip › Supplementary Figure S5.pdf]

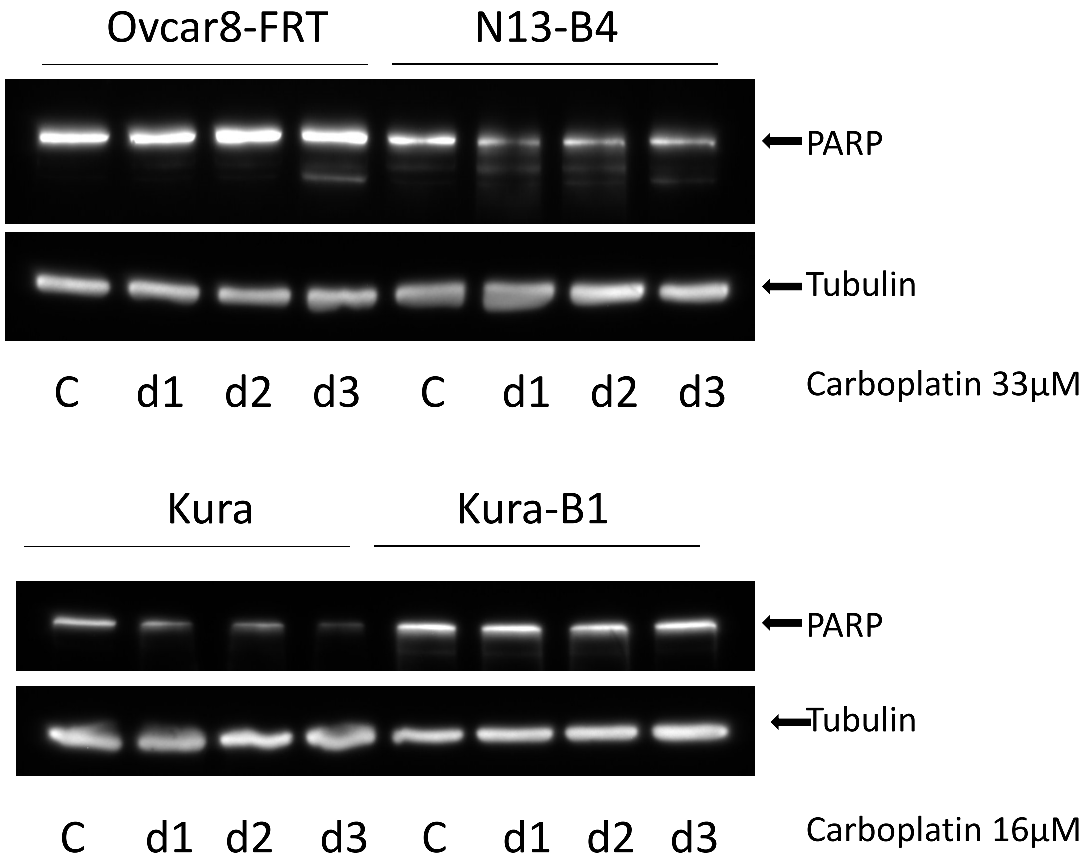

Supplementary Figure S5: PTPN13 increases sensitivity to apoptosis induced by platinum agents: OVCAR8-FRT and N13-B4 cells (Upper panel), wild type KURAMOCHI and Kura-B1 cells were treated with carboplatin for 1day (d1), 2 days(d2), 3 days(d3) or not treated(C). PARP expression was monitored by western blotting using anti-PARP antibodies. Equal loading was verified by re-probing with an anti-tubulin antibody.
